# Regulatory Hierarchies Controlling Virulence Gene Expression in *Shigella flexneri* and *Vibrio cholerae*

**DOI:** 10.3389/fmicb.2018.02686

**Published:** 2018-11-09

**Authors:** Matthew J. Dorman, Charles J. Dorman

**Affiliations:** ^1^Wellcome Sanger Institute, Wellcome Genome Campus, Hinxton, United Kingdom; ^2^Department of Microbiology, Moyne Institute of Preventive Medicine, Trinity College Dublin, Dublin, Ireland

**Keywords:** *Vibrio cholerae*, Shigella flexneri, cholera toxin, type 3 secretion system, virulence plasmid, chitin, biofilm, H-NS

## Abstract

Gram-negative enteropathogenic bacteria use a variety of strategies to cause disease in the human host and gene regulation in some form is typically a part of the strategy. This article will compare the toxin-based infection strategy used by the non-invasive pathogen *Vibrio cholerae*, the etiological agent in human cholera, with the invasive approach used by *Shigella flexneri*, the cause of bacillary dysentery. Despite the differences in the mechanisms by which the two pathogens cause disease, they use environmentally-responsive regulatory hierarchies to control the expression of genes that have some features, and even some components, in common. The involvement of AraC-like transcription factors, the integration host factor, the Factor for inversion stimulation, small regulatory RNAs, the RNA chaperone Hfq, horizontal gene transfer, variable DNA topology and the need to overcome the pervasive silencing of transcription by H-NS of horizontally acquired genes are all shared features. A comparison of the regulatory hierarchies in these two pathogens illustrates some striking cross-species similarities and differences among mechanisms coordinating virulence gene expression. *S. flexneri*, with its low infectious dose, appears to use a strategy that is centered on the individual bacterial cell, whereas *V. cholerae*, with a community-based, quorum-dependent approach and an infectious dose that is several orders of magnitude higher, seems to rely more on the actions of a bacterial collective.

## Introduction

**FIGURE 1:**
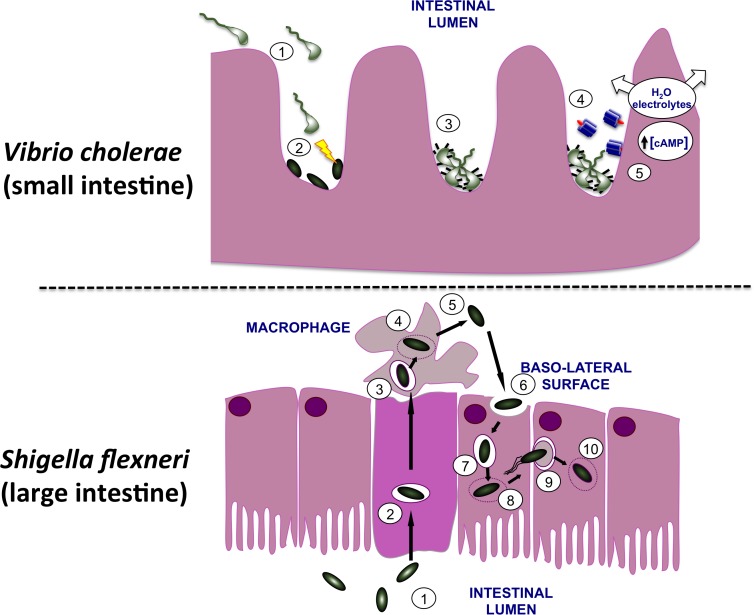
Infection of the human gut by *Vibrio cholerae* and *Shigella flexneri.* **(Top)** *V. cholerae* bacteria are shown moving from the lumen of the small intestine (1) to the surface of microvilli. Here they use their T6SS to eliminate commensal bacteria (2), creating a niche that they may then occupy on the gut wall. In response to signals that are characteristic of this niche, the bacteria produce TCP pili (3) and CT (4). The active moiety of the toxin A subunit enters the host cells and dysregulates adenylate cyclase, causing an increase in the cAMP concentration with a concomitant loss of water and electrolytes. This produces the watery diarrhea that is characteristic of cholera. **(Bottom)** *S. flexneri* bacteria (1) use their T3SS to invade the M cells of the lower gut epithelium (2). Following exit from these cells the bacteria encounter macrophage and are engulfed (3). The bacteria escape from the macrophage vacuole (4), triggering inflammatory cell death (5) and then invade gut epithelial cells via the basolateral surface (6). Once in the cell, *S. flexneri* escapes from the vacuole (7, 8), recruits and polymerizes host actin (9) and moves along the epithelial layer, cell by cell, escaping from each membrane double-envelopment in turn (10). The resulting damage to the epithelial layer produces an inflammatory response that amplifies the damage, causing the bloody diarrhea that characterizes dysentery.

*Shigella flexneri* and *Vibrio cholerae* are Gram-negative enteric pathogens of humans, causing the diarrheal diseases dysentery and cholera, respectively. Although both cause diarrheal diseases in humans and enter via the oral route, they differ in the site of infection, the mechanisms by which they cause damage to the host and in their infectious doses. Infections by *V. cholerae* leading to diarrheal disease arise from ingestion of contaminated food or water and affect the small intestine, with the bacterium remaining extracellular (Fu et al., 2018). *V. cholerae* uses a T6SS to eliminate competing members of the host microbiota at the site of infection(Zhao et al., 2018), colonizes the cleansed zone with the help of the TCP adhesin before producing CT to alter the physiology of adjacent epithelial cells (Figure 1). *V. cholerae* produces CT, a potent enterotoxin that is produced in response to environmental signals that are characteristic of the lumen of the human small intestine. CT binds to GM1 gangliosides on the epithelial layer (King and van Heyningen, 1973), enters the epithelial cell where it dysregulates human adenylate cyclase by ADP-ribosylation (Cohen and Chang, 2018), causing over-production of the second messenger cyclic adenosine monophosphate (cAMP) (Chen et al., 1971; Sharp and Hynie, 1971) (Figure 1). This in turn causes watery diarrhea characterized by a rapid loss of water and electrolytes that, if unchecked, can prove fatal to the patient (De Haan and Hirst, 2004).

In contrast, *S. flexneri* invades the M cells in the epithelium of the lower gut (Wassef et al., 1989; Perdomo et al., 1994). Next, *S. flexneri* undergoes transcytosis to the M cell pocket where it encounters and is engulfed by macrophage (Figure 1). The bacterium escapes from the macrophage vacuole by disrupting it, multiplies in the cytosol before inducing inflammatory cell death (Zychlinsky et al., 1992). The released *S. flexneri* bacteria invade nearby epithelial cells through their basolateral surfaces, promoting actin cytoskeleton rearrangements that result in engulfment of the bacteria (High et al., 1992). This produces bacteria-containing vacuoles that are soon ruptured (Weiner et al., 2016) with the released bacteria inducing actin polymerization at one pole of each microbial cell to achieve motility in the host cell cytosol (Mauricio et al., 2016). The bacterium is capable of moving from one cell to the next via clathrin-dependent endocytosis (Fukumatsu et al., 2012), escaping from the double-membrane-enclosed vacuole after each new invasion (Welch and Way, 2013). This damages the epithelium, induces an inflammatory response and results in the bloody diarrhea that is characteristic of dysentery (Ashida et al., 2015) (Figure 1).

This article will focus on the genetics of serogroup O1 and O139 *V. cholerae* biotype El Tor, which are responsible for the seventh cholera pandemic, and form a single phylogenetic lineage, 7PET (Domman et al., 2017). Although there are over 200 serogroups of *V. cholerae* (Shimada et al., 1994; Chapman et al., 2015), only serogroups O1 and O139 cause epidemic cholera. It should be noted that two biotypes of toxigenic *V. cholerae* O1, classical and El Tor, have been described, and there are differences in virulence gene expression between the two (Beyhan et al., 2006). Notably, classical strains of *V. cholerae* express virulence genes under standard laboratory conditions, and produce CT when cultured in rich media. By contrast, El Tor *V. cholerae* produce CT only when cultured under stringent *in vitro* growth conditions (Iwanaga and Yamamoto, 1985; Iwanaga et al., 1986). However, laboratory strains of both classical and El Tor *V. cholerae* have been used to dissect the regulatory systems described in this article.

*Shigella flexneri* and *V. cholerae* differ in the sizes of the doses required to initiate a successful infection. In the case of *S. flexneri*, the infectious dose can be as low as 10 cells whereas *V. cholerae* requires between 10^3^ and 10^8^ cells (Schmid-Hempel and Frank, 2007). It has been suggested that the presence of an extreme acid resistance (XAR) system in *S. flexneri* and its absence from *V. cholerae* may help to explain the difference in infectious dose: fewer *V. cholerae* bacteria will survive the transit through the low pH environment of the stomach, so a high infectious dose is necessary to establish an infection in the gut (Lund et al., 2014). The difference in infectious dose is also consistent with the important role that is played by cell-to-cell communication in *V. cholerae* compared with the more individualistic style that characterizes *S. flexneri* infection. In each pathogen, the genes encoding the principal virulence factors are only produced when the bacterium arrives at the correct location in the host. This indicates that the bacteria use physical and/or chemical information from the host to make a decision at the molecular level to produce or to repress production of virulence and colonization genes. In both examples, many genes have to be regulated collectively and their expression has to be finely controlled in space and time. In both cases, mobile genetic elements carry important virulence genes and their relationships with the bacterial chromosome have a significant influence on the stability of these genes and their expression.

## Horizontal Gene Transfer and Virulence in *S. flexneri* and *V*. *cholerae*

*Shigella flexneri* and *V. cholerae* both show evidence of an important role for horizontal gene transfer in the their evolution as pathogens. Many of the virulence genes are A + T-rich compared to the A + T content of the core genome, the transcription of these genes is silenced by the H-NS DNA binding protein and important genes are located on genetic elements that are either currently mobile and transmissible, or have been in the past (Buchrieser et al., 2000; Beloin and Dorman, 2003; Ayala et al., 2015a). In the case of *S. flexneri*, the majority of the virulence genes are carried on a large plasmid that is a genetic mosaic made from precursor plasmids and transposable elements (Buchrieser et al., 2000; Dorman, 2009) while many prominent virulence genes in *V. cholerae* are found on pathogenicity islands (Karaolis et al., 1998, 1999; Faruque et al., 2003) or in a filamentous bacteriophage (Waldor and Mekalanos, 1996). These features are explored in the following sections.

## *S. flexneri* Has Plasmid-Based Virulence Genes

**FIGURE 2:**
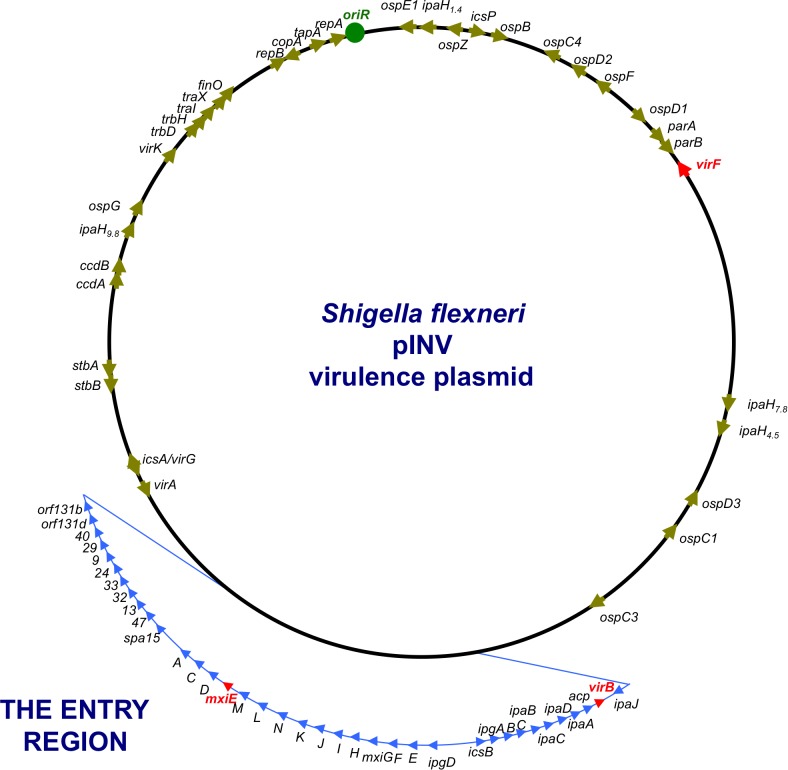
A genetic map of the pINV of *S. flexneri.* The plasmid is approximately 220 kbp in circumference and has a 31-kbp Entry Region (actually a pathogenicity island) where the principal virulence operons are located (light blue). Individual virulence genes can be seen around the plasmid, outside the Entry Region and others are located on the chromosome (not shown). The master regulatory genes *virF, virB*, and *mxiE* are shown in red.

*Shigella flexneri* uses a T3SS to inject effector proteins into host cells that produce the alterations to the actin cytoskeleton that are a characteristic of the invasive disease. Other virulence proteins help to create the actin comet tails that generate the movement of the bacteria through the host cytoplasm. Most of the principal T3SS-associated virulence genes are located on a large (213 kbp) plasmid called pINV, with those involved in the production of the T3SS being grouped in a so-called Entry Region (Figure 2) that is actually a pathogenicity island (Buchrieser et al., 2000; Lan et al., 2001; Dorman, 2009). The plasmid is capable of integrating into the chromosome, where expression of the virulence genes is silenced (Zagaglia et al., 1991; Colonna et al., 1995; Pilla et al., 2017). As virulence gene expression on the plasmid has been associated with its structural and genetic instability (Schuch and Maurelli, 1997), integration accompanied by silencing may be a strategy to ensure the vertical transmission of the virulence plasmid with the genes intact. Post-segregational killing by pINV-encoded toxin/anti-toxin systems also favors maintenance of the autonomously replicating plasmid by eliminating any plasmid-free cells that arise at cell division (Pilla et al., 2017; Pilla and Tang, 2018). The *S. flexneri* virulence plasmid is not self-transmissible (Sansonetti et al., 1982) but it contains genes encoding remnants of a *tra* transfer apparatus, suggesting that it has evolved from precursor plasmids that were mobile (Buchrieser et al., 2000). Although horizontal gene transfer via a mobile genetic element is not currently a feature of *S. flexneri* pathogenesis, it is central to that of *V. cholerae.*

## The Genes for Cholera Toxin Are on a Bacteriophage

The CT has an A_1_B_5_ structure and is encoded by the *ctxAB* operon (De Haan and Hirst, 2004). This operon is part of the CTXϕ filamentous phage genome (Waldor and Mekalanos, 1996) that usually inserts itself at the *dif* site on the larger chromosome of *V. cholerae* using the bacterial XerCD site-specific recombination system to catalyze insertion (Huber and Waldor, 2002; McLeod and Waldor, 2004; Das et al., 2013). Insertion is catalyzed by XerC alone and follows a pathway in which the plus strand of the virus genome is integrated and the complementary strand is generated by DNA replication (Val et al., 2005). *V. cholerae* has two chromosomes, with the smaller, second chromosome having many of the characteristics of a large plasmid (Val et al., 2016). This is interesting in the context of the comparison with *S. flexneri*, where the principal virulence genes are found on a large plasmid that replicates independently of the chromosome, almost like a mini-chromosome dedicated to causing disease. This *S. flexneri* plasmid is actually a mosaic of up to four plasmids and it has two functioning and one vestigial plasmid partition system (Buchrieser et al., 2000; Pilla and Tang, 2018). In an interesting example of the rather *ad hoc* and modular nature of bacterial gene control circuits, the VirB protein from the vestigial plasmid partitioning system has been integrated into the circuit that governs expression of the *Shigella* virulence phenotype (Adler et al., 1989; Watanabe et al., 1990; Taniya et al., 2003; Turner and Dorman, 2007).

**FIGURE 3:**
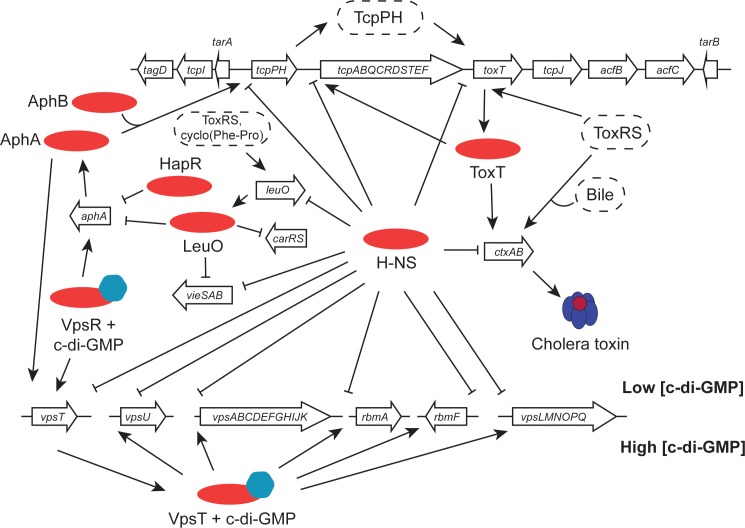
Virulence gene clusters in seventh pandemic *V. cholerae* share regulatory elements. Transcription promoters throughout the VPI-1 pathogenicity island and the *Vibrio* polysaccharide biosynthesis loci are silenced by the H-NS protein. The cholera toxin operon (*ctxAB*), located in the CTXϕ prophage at the *dif* site of chromosome I (classical strains have a second copy integrated at the equivalent site on chromosome II), is activated by the ToxT AraC-like protein and (under certain conditions) by ToxR. ToxR, together with TcpP, activates the *toxT* gene in VPI-1. It also positively regulates *leuO*, with LeuO performing important functions elsewhere in the genome, regulating *aphA* (in association with HapR), *carRS* and *vieSAB.* The *tcpPH* operon is under the positive control of the AphA and AphB proteins. At the Vps cluster, transcription is silenced by H-NS when c-di-GMP concentrations are low. When these rise, the VpsR activator switches on the *vpsT* regulatory gene and the VpsT protein activates the other chief transcription units. Transcription of *vpsT* is also stimulated by AphA.

Transcription of the *ctxAB* operon is under multifactorial control in response to a range of environmental signals (Figure 3). Among the agents of control is the H-NS nucleoid-associated protein, a DNA binding protein that is a silencer of transcription (Nye et al., 2000; Nye and Taylor, 2003; Stonehouse et al., 2011). H-NS has a preference for binding to A + T-rich DNA sequences and these are frequently a feature of genes that have been acquired by horizontal transfer. Since the *ctxAB* operon is part of an active bacteriophage, it is *de facto* a genetic element that is acquired in this way. H-NS has been studied best in the model organisms *Escherichia coli* and *Salmonella enterica* (Dorman, 2007). In *V. cholerae*, H-NS is also known as VicH, and it has approximately 50% amino acid sequence identity with its functional ortholog in *E. coli* (Tendeng et al., 2000; Cerdan et al., 2003). H-NS is a DNA bridging protein that also has the ability to polymerize along DNA, excluding other proteins (Dame et al., 2006; Dorman, 2007). Relief of H-NS-mediated transcription silencing can be achieved through myriad mechanisms (Stoebel et al., 2008; Dorman and Kane, 2009; Will et al., 2015). Several of these involve the intervention of other DNA binding proteins that antagonize the transcriptionally repressive activity of H-NS. In the case of the *ctxAB* operon, the ToxR protein binds to DNA using a wHTH and is an important H-NS antagonist. In fact, the primary biological function of ToxR appears to be opposition to the transcriptional silencing activity of H-NS (Kazi et al., 2016). ToxR appears to have relatively relaxed requirements for its DNA binding sites at the level of nucleotide sequence (Higgins and DiRita, 1996), possibly operating primarily via an indirect readout mechanism that relies on DNA shape (Dorman and Dorman, 2017). Among its most important regulatory targets is the *toxT* gene (Figure 3). The principal features of the ToxT regulatory protein are described in the following section.

## The *V. cholerae* AraC-Like ToxT Protein

ToxT is an AraC-like protein that binds to DNA using two HTH motifs, making it a member of a large family of bacterial transcription factors (Egan, 2002; Li et al., 2016). The *toxT* gene is located in VPI-1, a horizontally acquired genetic element that harbors many virulence genes (Karaolis et al., 1998, 1999; Faruque et al., 2003) (Figure 3). Prominent among these are the genes that encode the surface-expressed TCP. Here, the toxin in question is CT, with TCP playing an important role in the acquisition of CTXϕ by acting as the receptor for this filamentous phage (Waldor and Mekalanos, 1996) as well as being an important surface factor for autoaggregation and microcolony formation on the lining of the human gut (Taylor et al., 1987; Lim et al., 2010). Since TCP serves as the CTXϕ receptor, possession of VPI-1 is therefore necessary for *V. cholerae* to be lysogenised by CTXϕ and to become toxigenic. Given the patterns of CT and TCP production and their linked functions in the pathogenesis of *V. cholerae*, coordinated regulation of their respective genes is not unexpected. This coordination is achieved, in part, by the ToxT protein (DiRita et al., 1991; Higgins et al., 1992; Champion et al., 1997), which also regulates the transcription of its own gene (Brown and Taylor, 1995; Yu and DiRita, 1999) (Figure 3).

Like many AraC-like proteins, ToxT consists of two functional domains: its DNA binding domain is in the C-terminus of the protein and a ligand-binding domain and the dimerization functions are located in the N-terminus (Lowden et al., 2010). ToxT function is modulated by bile (Schuhmacher and Klose, 1999; Chatterjee et al., 2007) and oleic fatty acids are a component of bile; interestingly, *cis*-palmitoleic acid has been shown to bind to the N-terminus of ToxT in its crystallized form (Lowden et al., 2010). ToxT plays a dual role in transcription regulation, acting as an anti-repressor that overcomes gene silencing by H-NS as well as serving as a conventional transcription factor by recruiting RNA polymerase to target promoters (Yu and DiRita, 2002). The DNA sequences that are targeted by ToxT are A + T-rich, a feature that is shared by H-NS binding sites, making the two proteins natural antagonists. The ToxT protein binds to the promoter region of the CT operon and activates its transcription; the same operon is silenced by H-NS (Yu and DiRita, 2002; Stonehouse et al., 2011). ToxT is regarded as the primary protein regulator of *ctxAB* transcription, although ToxR can also play a role independently of ToxT under some growth conditions, e.g., when bile salts are present (Hung and Mekalanos, 2005) (Figure 3). ToxR was recognized early on as a central regulator of virulence gene expression in *V. cholerae*, transmitting information about the composition of the external environment to its target genes (Miller et al., 1987). It has become clear that ToxR is part of a much more complex regulatory cascade with the *toxT* gene as one of its key targets (see the following section).

## The ToxRS/TcpPH Partnership in *V. cholerae*

The ToxRS/TcpPH regulatory protein partnership transmits information about growth medium composition, pH, osmolarity, and temperature to the *V. cholerae* virulence regulon (Taylor et al., 1987; Miller and Mekalanos, 1988; Peterson and Mekalanos, 1988; Thomas et al., 1995; Carroll et al., 1997). ToxR is a cytoplasmic-membrane-associated DNA binding protein that exists in a complex with ToxS, another transmembrane protein (Ottemann and Mekalanos, 1996). The genes that encode these two proteins form an operon that is a component of the core genome of *V. cholerae* and is expressed constitutively (Miller et al., 1989). The N-terminus of the ToxR protein contains the wHTH DNA binding domain while the C-terminus is located in the periplasm of the bacterium (Miller et al., 1987; DiRita and Mekalanos, 1991). It was noticed early on that ToxR forms either ToxR–ToxR homodimers or ToxR-ToxS heterodimers (Ottemann and Mekalanos, 1996). ToxR undergoes regulated intramembrane proteolytic degradation and is protected from this by ToxS and by an intramolecular disulphide bond between two cysteines in the ToxR periplasmic domain (Fengler et al., 2012; Midgett et al., 2017). Exposure to bile salts or other detergents enhances ToxR–ToxS contact and increases the influence of ToxR on gene expression: the stability of ToxR declines as the bacterium makes its immediate environment more alkaline in stationary phase, disrupting ToxR’s protective interaction with the ToxS protein (Midgett et al., 2017).

Paralogous counterparts to ToxR and ToxS, known as TcpP and TcpH, are encoded by genes on VPI-1 (Figure 3). The AphA and AphB transcription factors, encoded by the ancestral part of the genome, act cooperatively at the *tcpPH* promoter to activate transcription (Kovacikova and Skorupski, 2001). The cAMP-Crp complex also regulates this promoter (Kovacikova and Skorupski, 2001). TcpP and TcpH form a regulatory complex that is analogous to the ToxRS one, and they also regulate the transcription of the *toxT* gene (Häse and Mekalanos, 1998; Krukonis et al., 2000). Like ToxR, the TcpP transcriptional activator is subject to regulated intramembrane proteolysis, making it unstable (Teoh et al., 2015). Its TcpH partner protects TcpP from such degradation (Beck et al., 2004). The presence of a disulphide bond within the periplasmic domain of TcpP is important for the protection of both TcpP and TcpH from degradation (Morgan et al., 2015).

ToxR and TcpP cooperate in the positive regulation of *toxT* (Morgan et al., 2011) and single-molecule-tracking experiments show that ToxR recruits TcpP to the *toxT* promoter (Haas et al., 2015) (Figure 3). The two proteins make contact through wing-wing interactions in their wHTH DNA binding domains (Krukonis and DiRita, 2003; Goss et al., 2010). Once ToxT is produced, it activates the other genes in its regulon, including the genes for the TCP, the accessory colonization factor (ACF), aldehyde dehydrogenase, and the CT operon itself (DiRita et al., 1991). ToxT also induces the production of the small regulatory RNA TarB, encoded within the *tcp* gene cluster (Figure 3). When made stable by the Hfq RNA chaperone, TarB down-regulates the VspR transcription repressor resulting in derepression of virulence genes in the *Vibrio* seventh pandemic pathogenicity island VSP-1 in 7PET *V. cholerae* (Davies et al., 2012). The expression of these genes produces increased intracellular concentrations of signaling molecules such as cyclic-GMP/cyclic-AMP and possibly c-di-GMP/c-di-AMP, altering the metabolism of the bacterium and its chemotactic behavior (Davies et al., 2012; Dorman, 2015; Severin et al., 2018) (Figure 3). TarB also post-transcriptionally down-regulates TcpF production from VPI-1: here, Hfq does not seem to be required and the production of the sRNA is enhanced during anaerobic growth (Bradley et al., 2011) (Figure 3).

**FIGURE 4:**
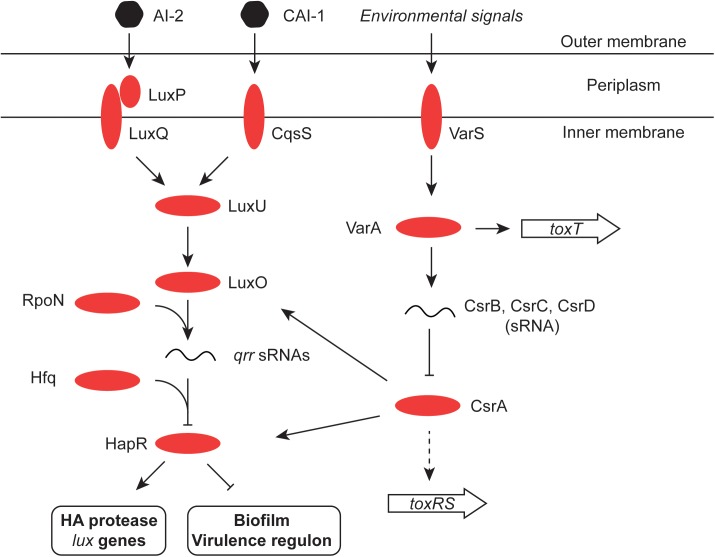
Quorum sensing in *V. cholerae.* The signaling molecules CAI-1 and AI-2 are detected by the cytoplasmic membrane protein CqsS and the periplasmic binding protein LuxP, respectively. LuxP interacts with LuxQ which, like CqsS, interacts in turn with LuxU in the cytoplasm. LuxU activates the LuxO regulatory protein and this promotes expression of the Qrr sRNA from an RpoN (σ^54^)-dependent promoter. Aided by the RNA chaperone Hfq, Qrr inhibits the production of the HapR regulatory protein (an activator of the HA protease and the *lux* genes, and an inhibitor of the virulence regulon and the biofilm production). LuxO production is also under the control of the global regulatory protein CsrA, which in turn is inhibited by the redundant sRNA molecules CsrB, C, and D. CsrA also influences production of the HapR transcription factor directly, as well as indirectly through LuxO (Tsou et al., 2011). The two-component VarS–VarA system responds to environmental and quorum-sensing signals and promotes the production of the Csr sRNAs. It has also been reported to modulate the expression of the ToxT protein (Jang et al., 2011). Finally, CsrA may directly promote production of ToxR in response to the amino acids asparagine, arginine, glutamate, and serine (Mey et al., 2015).

**FIGURE 5:**
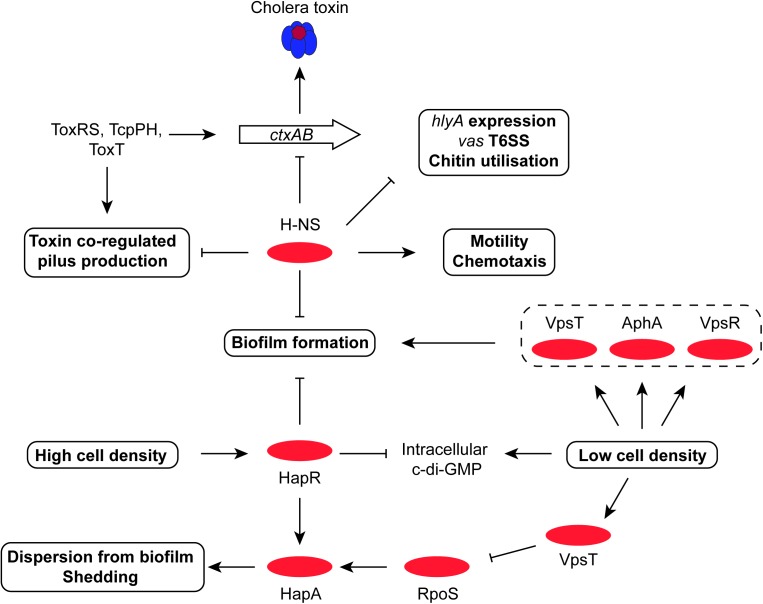
The central roles of HapR and H-NS in biofilm formation, motility, T6SS and chitin utilization. The H-NS protein plays a positive role in *V. cholerae* motility, as it does in other Gram-negative bacteria. It represses expression of genes for production of T6SS, virulence, chitin utilization, and biofilm. At high cell density, the HapR transcription factor also represses biofilm formation while promoting dispersion of bacteria from biofilm and shedding; it also promotes the production of the stress and stationary phase sigma factor RpoS (σ^38^). RpoS and HapR cooperate to promote the production of the HapA protease, which erodes biofilm. The VpsR-and-c-di-GMP-dependent VpsT regulatory protein down-regulates RpoS, helping to maintain the dominance of RpoD (σ^70^) in metabolically-active cells.

H-NS plays an important role in the control of gene expression in the context of adaptation to environments outside the human host (Ayala et al., 2017) (Figure 3). It is also required for *V. cholerae* motility (Ghosh et al., 2006; Silva et al., 2008). Apart from the *ctxAB* operon, most of the genes that are silenced by H-NS are located in VPI-1 on chromosome I and in a 125-kb super-integron located on chromosome II; others include the *hlyA* hemolysin/cytolysin, the *vas* major type VI secretion operon and the *vps-rbm* biofilm and chitin utilization genes (Ayala et al., 2015a,b; Wang et al., 2015; Zamorano-Sánchez et al., 2015; Kazi et al., 2016) (Figures 3–5).

## The AphA AphB Regulon

AphA is a wHTH transcription factor (De Silva et al., 2005) that assists the LysR-like protein AphB to bind to the *tcpPH* promoter (Kovacikova and Skorupski, 2001; Kovacikova et al., 2004) (Figure 3). AphA represses the transcription of the *V. cholerae* genes that synthesize acetoin and hence helps to manage the pH of the bacterial cytoplasm (Kovacikova et al., 2005). AphA activates the expression of the gene encoding VpsT, a LuxR-like biofilm regulator of biofilm formation (Yang et al., 2010) (Figure 3). The VpsR regulatory protein activates production of VpsT in the presence of high c-di-GMP; VpsR also activates the gene encoding AphA under these conditions (Ayala et al., 2015a,b; Dorman, 2015; Zamorano-Sánchez et al., 2015) (Figure 3). These VpsR/T-AphA connections link the virulence and biofilm gene clusters intimately at the level of cross regulation.

HapR, the chief quorum-sensing regulator of *V. cholerae*, is known to repress the transcription of the *tcpPH* gene and hence the virulence regulon (Zhu et al., 2002). HapR binds to, and represses, the *aphA* promoter when the bacterial culture is at high density, preventing activation of *tcpPH* by AphA (Kovacikova and Skorupski, 2002; Kovacikova et al., 2003). This link makes the virulence cascade responsive to quorum sensing (Figures 3, 4). The HapR and AphA proteins play reciprocal roles in the response of *V. cholerae* to cell density: AphA operates at low cell density and HapR takes over the role of master regulator at high cell density. At low cell density, AphA production is upregulated by sRNAs and then AphA and Hfq-dependent sRNAs repress production of HapR (Lenz et al., 2005; Lenz and Bassler, 2007; Rutherford et al., 2011; Basu et al., 2017) (Figure 4). These (redundant) sRNA molecules are four Quorum Regulatory RNAs (Qrr) and they act with Hfq to repress production of HapR. In parallel, the production of the CsrB, CsrC, and CsrD sRNAs (also redundant) is under the control of the *V. cholerae* two-component system VsrS/VsrA (Lenz et al., 2005) (Figure 4). These Csr sRNAs act on the CsrA global regulatory protein to activate production of the Qrr sRNAs via the LuxO quorum-sensing protein, in an RpoN-dependent manner (Lenz et al., 2005). At high cell density, HapR acts at *aphA* by antagonizing its activation by the leucine-responsive regulatory protein, Lrp, and the NtrC-like biofilm regulator, VpsR (Lin et al., 2007) (Figure 3). In *Shigella*, CsrA has a poorly defined impact on the virulence phenotype through effects on expression of the *virF* and *virB* regulatory genes, implicating carbon metabolism as an important determinant of *Shigella* virulence (Gore and Payne, 2010). Quorum sensing seems to play a role in production of the *S. flexneri* T3SS, affecting in particular expression of the *virB* regulatory gene. T3SS production is maximal in cells entering the stationary phase of growth, a stage when bacterial cell density is maximal during growth in laboratory culture. However, the details of the signaling cascade remain obscure (Day and Maurelli, 2001).

AphB plays an important role in *V. cholerae* pH homeostasis by regulating, among other genes, the expression of *cadC* (Kovacikova et al., 2010). The CadC protein shares with ToxR and TcpP the properties of being a cytoplasmic-membrane-associated transcription regulator (Merrell and Camilli, 2000). Among its targets is the *cadBA* operon encoding a lysine decarboxylase (CadA) and a lysine/cadaverine antiporter (CadB). CadA converts lysine to cadaverine and CadB transports cadaverine out of the cell, exchanging it for lysine (Merrell and Camilli, 1999). Interestingly, *cadA* has been identified as an anti-virulence gene that is deleted in *Shigella* species: it has been suggested that since cadaverine inhibits *Shigella* enterotoxin activity, loss of *cadA* by deletion enhances the virulence of the pathogen (Maurelli et al., 1998).

The *cadC* gene in El Tor *V. cholerae* is repressed by the LysR-like wHTH transcription factor LeuO (Ante et al., 2015a), a protein with wide-ranging effects on transcription in Gram-negative bacteria (Shimada et al., 2011; Dillon et al., 2012; Guadarrama et al., 2014; Dorman and Dorman, 2017). ToxR derepresses the H-NS-silenced *leuO* gene (Bina et al., 2016) and it also mediates the transcription of *ompU*, a gene encoding an outer membrane porin that confers resistance to bile stress (Ante et al., 2015b) (Figure 3). LeuO represses the *carRS* operon that controls the remodeling of lipid A for the elaboration of cationic antimicrobial peptide resistance (Bina et al., 2016). In classical strains of *V. cholerae*, LeuO is a co-repressor, with H-NS of *vieSAB* transcription, an operon that modulates biofilm formation and cell motility (Ayala et al., 2018). In contrast, El Tor biotype strains produce very little LeuO. The *vieSAB* operon is almost silent in the El Tor biotype due to repression by the quorum-sensing regulator, HapR, a protein that is reported to be absent from classical biotype strains (Wang et al., 2015). The emerging picture is one in which H-NS and LeuO cooperate to repress *vieSAB* in classical strains and H-NS and HapR achieve the corresponding effect in El Tor organisms (Ayala et al., 2018). Consistent with this role, *leuO* mutants exhibit reduced levels of biofilm production (Moorthy and Watnick, 2005). LeuO, whose production is enhanced by ToxR, feeds back negatively onto *aphA* transcription, leading to downregulation of the ToxR regulon with reductions in CT and TCP production (Figure 3). The signal for the ToxR enhanced transcription of *leuO* is cyclo(Phe-Pro) (Bina et al., 2013).

## Signals Affecting CT Production: Role of the Stringent Response

AphB has also been linked to the anaerobic response in *V. cholerae* (Kovacikova et al., 2010). Its dimerization and activity in regulating the expression of *tcpPH* are enhanced by anaerobiosis. A cysteine amino acid has been shown to be essential for the anaerobic enhancement, indicating that AphB uses a thiol-based switch to respond to oxygen limitation (Liu et al., 2011). Anaerobiosis and the stringent response are linked in enhancing the production of CT (Lee et al., 2012). When grown anaerobically using trimethylamine *N*-oxide (TMAO) as a terminal electron acceptor, *V. cholerae* exhibits enhanced production of CT. The bacteria also produce the alarmone guanosine tetraphosphate (ppGpp) whereas mutants deficient in ppGpp production are also impaired in CT production. Similarly, mutants lacking DksA, the protein that works synergistically with ppGpp at RNA polymerase, are also impaired in CT production (Oh et al., 2014). The stringent response is a global reaction to nutritional stress that is usually triggered by an accumulation of uncharged tRNAs that indicate a shortage of one or more amino acids. Bacteria experiencing the stringent response accumulate ppGpp and reprogramme RNA polymerase so that genes expressing components of the translational machinery are downregulated. The enzyme RelA produces pppGpp from ATP and GTP and then converts it to ppGpp; SpoT hydrolyses ppGpp (although SpoT has a synthesis role too) (Dalebroux et al., 2010; Dalebroux and Swanson, 2012). In *V. cholerae* the RelV enzyme synthesizes ppGpp in the absence of RelA and SpoT when the bacterium is experiencing glucose or fatty acid starvation (Das et al., 2009). All three synthases are involved in regulating *V. cholerae* biofilm production: *vpsR* transcription requires all three while *vpsT* requires RelA (He et al., 2012). *V. cholerae* also has a GTP-binding protein called CgtA that modulates the function of SpoT, repressing the stringent response by keeping ppGpp levels low (Raskin et al., 2007). DksA regulates the production of the hemagglutinin/protease (HAP) that is involved in the shedding stage of the *V. cholerae* infection (Basu et al., 2017). HAP production is controlled by HapR and RpoS with DksA controlling HapR production positively at the transcriptional and post-transcriptional levels. The post-transcriptional regulation involves Fis-dependent Quorum Regulatory RNAs (Qrr) sRNAs and the levels of these regulatory RNA molecules are strongly reduced in the absence of DksA (Lenz et al., 2005; Lenz and Bassler, 2007). Furthermore, DksA is required for normal levels of RpoS production in stationary phase *V. cholerae* cells (Basu et al., 2017). The counterpart to CgtA in *E. coli* (and *Shigella*) is the evolutionarily highly conserved Obg GTPase (Dewachter et al., 2016). Obg has been described as a cell cycle protein that can arrest chromosome replication, a factor required for stress response and for ribosome assembly. In association with ppGpp it determines persistence through the stochastic induction of a state of dormancy and antibiotic tolerance during periods of nutrient stress (Verstraeten et al., 2015; Gkekas et al., 2017). In *S. flexneri*, the DksA protein and ppGpp are required for production of the Hfq RNA chaperone (Sharma and Payne, 2006) a protein that controls the level of the central virulence regulator VirB (Mitobe et al., 2008, 2009). Loss of DksA impairs the intercellular spreading of *S. flexneri*, with *dksA* mutants having the IcsA protein that is required for actin-dependent motility mislocated on the cell surface (Mogull et al., 2001). The stringent response operates by reprogramming the activity of RNA polymerase in response to amino acid starvation. RNA polymerase is also reprogrammed in response to stress by the replacement of the housekeeping sigma factor RpoD by a stress-specific sigma factor, with RpoS being a prominent example.

## General Response to Stress and the Role of the RpoS Sigma Factor

Many commensal and pathogenic bacteria respond to stress and/or the cessation of growth by reprogramming RNA polymerase by replacing the RpoD sigma factor with RpoS (Klauck et al., 2007; Schellhorn, 2014). The role of the RpoS sigma factor in the expression of virulence has been investigated more intensively in *V. cholerae* than in *S. flexneri.* In *S. flexneri*, much of the focus has been on the link between RpoS and resistance to low pH stress. The discovery that *Shigella* and some pathogenic *E. coli* strains have a regulator called IraL that stabilizes RpoS in the absence of stress may reveal infection-related RpoS roles in *Shigella* that have yet to be explored (Hryckowian et al., 2014). In terms of where RpoS sits in the *V. cholerae* regulatory hierarchy, it is useful to note that transcription silencing by H-NS may be less effective in *V. cholerae* at RpoS-dependent promoters (Wang et al., 2012), although it is not clear if this is an entirely due to an intrinsic property of the RpoS sigma factor or if some other feature, such as the topology of the promoter DNA during periods of RpoS availability, might play a role. Also, RpoS in *V. cholerae* enhances the production of IHF, with knock-on effects for IHF-dependent processes (Wang et al., 2012). Early work with *V. cholerae* showed that loss of RpoS production makes the bacterium sensitive to a variety of environmental stresses *in vitro* (Yildiz and Schoolnik, 1998) and interferes with its ability to colonize the mouse small intestine (Merrell et al., 2000). The sigma factor is also involved in the latter stages of infection when the bacterium detaches from the epithelial surface. Here, the so-called mucosal escape step requires RpoS with the sigma factor contributing to the expression of genes involved in motility and chemotaxis, functions that are important for the subsequent dispersal of the pathogen (Nielsen et al., 2006; Ringgaard et al., 2015). Motility genes have a complex relationship with the virulence phenotype, with mutants deficient in specific flagellar hierarchy regulators displaying altered production of virulence factors including CT, TCP and the T6SS (Syed et al., 2009). *V. cholerae’*s re-entry to an aquatic environment may also be RpoS-dependent. The sigma factor is required for the production of the chitinase that renders *V. cholerae* both competent for the uptake of foreign DNA and which is needed for the growth of the bacterium on otherwise insoluble chitin (Dalia, 2016). This RpoS-competence link is independent of any quorum-sensing link to natural transformation by foreign DNA (Dalia, 2016). HapR and quorum sensing have been reported to enhance the production of RpoS (Joelsson et al., 2007) and RpoS plays a positive role in the expression of the HapR-dependent *V. cholerae hapA* gene, encoding a hemagglutinin/protease (Silva and Benitez, 2004) (Figure 4). This enzyme plays an important part in the bacterial shedding phase that occurs toward the end of the infection (Basu et al., 2017). Transcription of the *rpoS* gene is repressed by the VpsT transcription factor, in association with the second messenger cyclic-di-GMP, when *V. cholerae* is within biofilm (Wang et al., 2014) (Figure 5). While it has been appreciated for some time that RpoS (and quorum sensing) are important influences in determining entry into a biofilm, it is now becoming clear that RpoS and quorum sensing are also important for *V. cholerae* dispersion from biofilm (Singh et al., 2017).

## Chitin and Gene Regulation in *V. cholerae*

**FIGURE 6:**
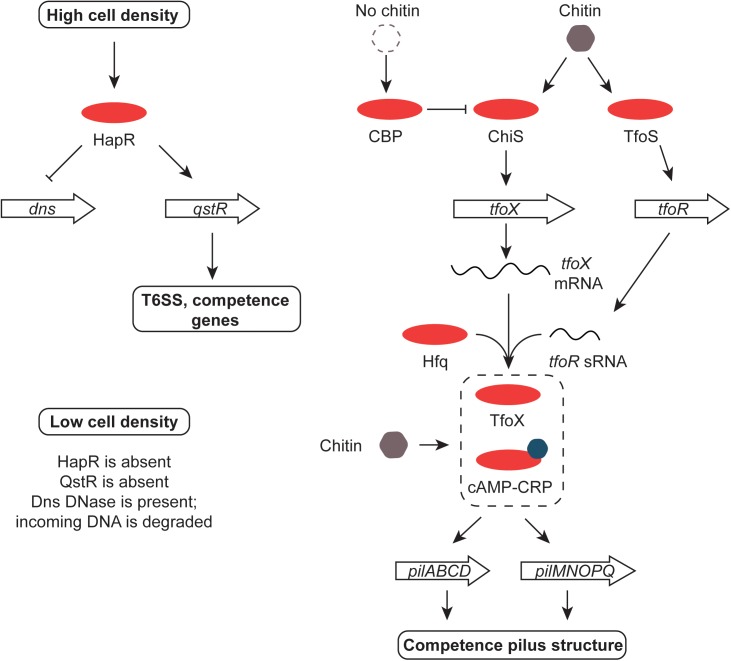
Chitin, HapR, QstR, and competence. At high cell density, HapR represses production of the Dns DNase, an enzyme that degrades foreign DNA. It also promotes the production of the QstR regulator, switching on the T6SS and genes for competence. The former kills nearby cells, releasing DNA for uptake by competent *V. cholerae.* The genes encoding the pilus that forms the DNA uptake system are up-regulated in response to chitin by the cAMP-Crp complex and the TfoX regulatory protein. The *tfoX* gene is activated by ChiS in response to chitin and *tfoX* mRNA is translated efficiently in the presence of the Hfq-dependent TfoR sRNA. The switch is reset in the absence of chitin by the CBP protein. Production of the TfoR sRNA is promoted by the TfoS regulator in response to chitin. When cell density is low, HapR and QstR are absent, allowing the Dns enzyme to be produced and foreign DNA to be degraded.

Chitin transport and metabolism depend on the *chb* operon in *V. cholerae.* The cell division protein SlmA binds to *chb* and regulates its expression, possibly in association with an unknown transcription factor that is under the control of the ChiS sensor-kinase (Klancher et al., 2017). Cbp is a periplasmic chitin binding protein that regulates ChiS negatively when chitin is absent; binding chitin by Cbp relieves repression (Li and Roseman, 2004) (Figure 6). TfoS is an independent chitin sensor that activates the transcription of the *tfoR* gene that encodes an sRNA, TfoR (Dalia et al., 2014; Yamamoto et al., 2014). This Hfq-dependent sRNA positively controls the translation of *tfoX* mRNA with the TfoX protein, in turn, activating genes involved in foreign DNA uptake in competent cells (Yamamoto et al., 2011) (Figure 6). The *tfoX* gene is also regulated at the level of transcription by the cAMP-Crp complex (Wu et al., 2015). This is consistent with the roles of cAMP-Crp and chitin in the establishment of competence in *Vibrio* spp. (Blokesch, 2012). Chitin makes *V. cholerae* competent for DNA uptake by transformation, allowing it to influence the evolution of the bacterium by horizontal gene transfer (Meibom et al., 2005; Le Roux and Blokesch, 2018). The fate of DNA taken up by *V. cholerae* that has been made competent by chitin depends on cell density and quorum sensing. At low cell density the HapR regulator is present in low abundance, allowing the Dns nuclease to accumulate and to degrade the incoming DNA extracellularly or in the periplasm (Blokesch and Schoolnik, 2008; Seitz and Blokesch, 2014). At high cell density, HapR is abundant and production of the nuclease is repressed. Under these conditions the TfoX-dependent competence system imports the DNA (Seitz and Blokesch, 2013) (Figure 6). In addition to its roles in controlling competence, the cAMP-Crp complex also controls the virulence regulon at the *tcpPH* promoter (Kovacikova and Skorupski, 2001) and both the integrase gene promoter and the main promoter for gene cassette transcription in the superintegron found on chromosome II of *V. cholerae* (Krin et al., 2014). The gene that encodes the outer membrane porin protein OmpT requires cAMP-Crp for expression; *ompT* is the only member of the ToxR regulon known to be repressed by ToxR and it acts by disrupting the positive influence of the cAMP-Crp complex at the *ompT* promoter (Caiyi et al., 2002).

## Gene Silencing and Anti-Silencing in *S. flexneri*

**FIGURE 7:**
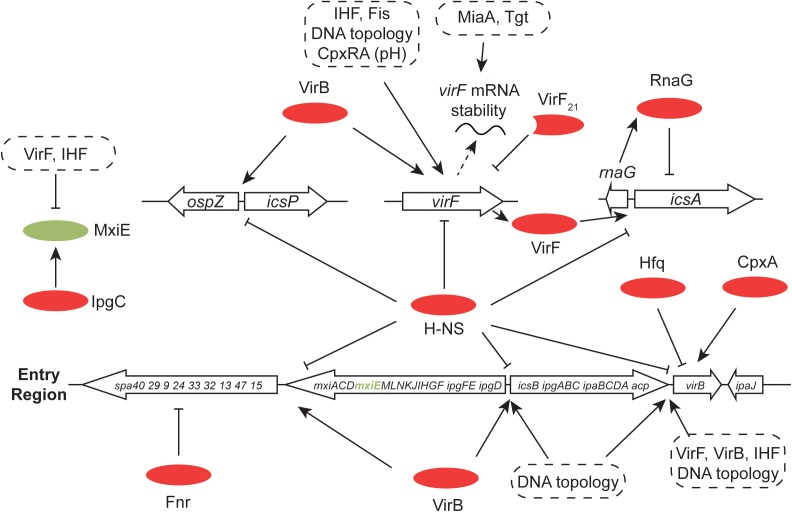
Global control of the *S. flexneri* virulence system. The 31-kbp Entry Region, consisting of three large operons, is indicated. All of the main transcription promoters in this region are repressed by H-NS and this is reversed by the Entry-Region-encoded VirB anti-repressor. Production of VirB depends on the VirF AraC-like protein (encoded by pInv), the correct degree of DNA negative supercoiling and the chromosomally-encoded IHF protein. VirB autoregulates its own gene positively and cross-regulates positively the gene for *virF*. VirB is also subject to Hfq-dependent post-transcriptional control at the level of mRNA stability. In addition to VirB, production of VirF requires IHF, an appropriate degree of DNA negative supercoiling, the chromosomally encoded Fis protein and the chromosomally encoded pH-responsive CpxRA two-component regulatory system. VirF is controlled post-transcriptionally by the tRNA-modifying MiaA and Tgt factors. The truncated form of VirF, VirF_21_, acts as an antagonist of full-length VirF production. MxiE is another AraC-like protein with multiple gene targets on pInv (and on the chromosome) whose activity is modulated by the IpgC co-activator and the anti-activators OspD1 and Spa15. The Fnr anaerobic regulator makes negative regulatory inputs at *spa32* and *spa33.* The VirF-dependent *icsA* gene, repressed transcriptionally by H-NS and RnaG, is shown, together with the divergently-transcribed-and-H-NS-repressed, VirB-activated *icsP* and *ospZ* genes.

The H-NS protein plays a general role in silencing transcription throughout the virulence regulon of *S. flexneri* (and enteroinvasive *E. coli*) (Dorman et al., 2001; Beloin and Dorman, 2003) (Figure 7). Like the *V. cholerae* system, the *S. flexneri* regulon uses an AraC-like protein to overcome H-NS-mediated transcription silencing (Dorman, 1992; Porter and Dorman, 2002). The VirF protein performs approximately an equivalent role to ToxT and is encoded by *virF*, a regulatory gene on the virulence plasmid that lies outside the Entry Region (Adler et al., 1989; Porter and Dorman, 1997b, 2002; Di Martino et al., 2016a). It is closely related to the Rns protein of enterotoxigenic *E. coli* and the two proteins are at least partly functionally interchangeable (Porter et al., 1998). Transcription activation of *virF* involves a complicated process in which H-NS-mediated silencing is overcome by an adjustment to the local DNA structure at the *virF* promoter (Stoebel et al., 2008). This adjustment is triggered by a shift in temperature to 37°C from a lower value. This temperature is one of the environmental signals, along with appropriate values of pH and osmolarity that are characteristic of the human intestine (Di Martino et al., 2016a). It involves a change in the bend angle of a region of A + T-rich DNA that widens the space between the arms of the bend, possibly compromising the integrity of a DNA-H-NS-DNA bridge, an event that is exacerbated by a displacement of the center of the bend that disrupts the parallel placement of the DNA bend arms (Prosseda et al., 2004). This remodeling event does not require the intervention of proteins, although the Fis and IHF nucleoid-associated proteins play a positive role in *virF* transcription activation and the VirB protein also makes a direct and positive contribution (Porter and Dorman, 1997a; Prosseda et al., 2004; Kane and Dorman, 2012). DNA remodeling to activate *virF* transcription is consistent with the known role of DNA negative supercoiling in modulating the expression both of *virF* (Durand et al., 2000) and the wider *S. flexneri* virulence system (Dorman et al., 1990; Ni Bhriain and Dorman, 1993; Dorman, 1995; McNairn et al., 1995). There is also a positive regulatory role for the CpxA/CpxR two-component system (that transmits information about ambient pH) in the activation of *virF* expression (Nakayama and Watanabe, 1995, 1998; Marman et al., 2014). CpxA has also been described as influencing positively the stability of *invE/virB* mRNA (Mitobe et al., 2005).

## Control of VirF Production in *S. flexneri*

The *virF* transcript is translated into two proteins: a major form known as VirF_30_ (which has a molecular mass of 30 kDa) and VirF_21_, lacking the N-terminal segment of the full-length protein (Di Martino et al., 2016b). While VirF_30_ is responsible for the activation of the virulence regulon, VirF_21_ is a negative autoregulator of *virF* expression. The shorter protein is translated from a leaderless mRNA called llmRNA that is transcribed from a separate promoter that is internal to *virF*: the function of VirF_21_ may be to restrict production of VirF to environments where *S. flexneri* will benefit from expression of the full virulence regulon (Di Martino et al., 2016b). Production of the VirF protein is reduced in mutants with deficiencies in tRNA modification: specifically in *miaA* and *tgt* mutants (Durand et al., 2000). This reduction in VirF production has a negative impact on *S. flexneri* virulence and results from poor translation of the *virF* mRNA. This negative effect can be offset by the presence of methionine and arginine in combination, or by adding putrescine to the growth medium (Durand and Björk, 2003). These observations indicate the extent to which VirF production is keyed into central metabolism in *S. flexneri*.

A striking feature of the *S. flexneri* virulence gene regulatory cascade is that VirF does not regulate all of the main structural genes directly. Its direct influence is restricted to the ‘intracellular spread’ gene *icsA* (also known as *virG*) a small RNA antagonist of *icsA* transcription called RnaG, and to an intermediate regulatory gene called *virB*. The H-NS protein silences each of these genes (Tran et al., 2011) (Figure 7). The *icsA* gene product is localized to one of the poles of the *S. flexneri* cell where it promotes the polymerization of actin that, in turn, creates the characteristic comet-tail-like structures that propel the bacterium through the host cell cytoplasm (Suzuki et al., 1996, 1998).

## The Unusual VirB Regulatory Protein in *S. flexneri*

The *virB* gene, known as *invE* in *S. sonnei* (Watanabe et al., 1990), encodes a protein with strong amino acid sequence similarity to plasmid segregation proteins of the ParB family (Adler et al., 1989; Beloin et al., 2002; McKenna et al., 2003; Taniya et al., 2003; Turner and Dorman, 2007). VirB acts between VirF and most of the virulence gene promoters of the *S. flexneri* virulence regulon, carrying out the role of an intermediate regulator (Figure 7). VirB has a requirement for a specific *parS-*like DNA sequence at its binding sites and its mode of action consists of remodeling H-NS-DNA complexes to eliminate the transcription silencing activity of the H-NS protein (Taniya et al., 2003; Turner and Dorman, 2007). The *icsB* gene is first in an operon that encodes components of the *S. flexneri* T3SS and it is silenced by H-NS (Beloin and Dorman, 2003). The counter-silencing mechanism used at the *icsB* promoter seems to consist of a combination of DNA wrapping and VirB protein polymerization (Turner and Dorman, 2007). VirB antagonism of H-NS-mediated silencing can act over considerable distances, as at the *icsP* promoter (Castellanos et al., 2009). The IcsP (SopA) protein is a protease that deactivates IcsA, the cell-pole-located actin-tail-polymerizing protein (Fukuda et al., 1995; d’Hauteville et al., 1996; Shere et al., 1997; Wing et al., 2004). The *icsP* promoter is silenced by H-NS and VirB acts there as an anti-repressor; contact between the counteracting proteins may involve DNA dependent protein polymerization *in vivo* (Weatherspoon-Griffin et al., 2018). The *ospZ* promoter is also controlled by a VirB/H-NS-dependent silencing/anti-silencing mechanism (Basta et al., 2013). The OspZ protein is secreted by the *S. flexneri* T3SS to manipulate the host inflammatory response (Zurawski et al., 2008; Zhang et al., 2016). The *ospZ* gene is located immediately upstream of *icsP* and is transcribed in the opposite direction. The two genes share important *cis*-acting regulatory sites that are targets for VirB when acting in its capacity as an anti-repressor (Basta et al., 2013). A similar arrangement is found between the *icsB* and *ipgD* genes whose promoters are silenced by H-NS and derepressed by VirB (Porter and Dorman, 1997a; Taniya et al., 2003; Turner and Dorman, 2007).

Early work showed that the *virB* gene is repressed by H-NS and regulated positively by VirF (Tobe et al., 1993) (Figure 7). Although the activation of *virB* transcription depends on the presence of VirF, the presence of this AraC-like protein is not sufficient: a temperature of 37°C is also required. This thermal requirement can be dispensed with if the topology of the DNA in the vicinity of the *virB* promoter is manipulated appropriately, an observation that supports a model in which the positive role of VirF is contingent on the conformation of the DNA on which it acts (Tobe et al., 1995). Consistent with this hypothesis is the finding that negative DNA supercoils, generated by a divergently transcribing promoter, activate the *virB* promoter in the presence of VirF at the normally non-permissive temperature of 30°C (Tobe et al., 1995). The *virF* and *virB* genes represent the principal sites for thermal sensing in the *S. flexneri* virulence gene cascade. Once the VirB protein is produced, the genes under its control will be derepressed as H-NS-mediated transcription silencing is progressively relieved. This can be shown by producing the VirB protein ectopically, e.g., using an arabinose-inducible promoter in bacteria growing at 30°C, a temperature that is normally non-permissive for expression of the virulence regulon. Here, the level of transcription of the virulence genes corresponds to the level of arabinose-induced VirB protein that is present in the cell (Beloin and Dorman, 2003). The *virB/invE* transcript becomes unstable at 30°C (Mitobe et al., 2008) or if the osmolarity of the growth medium declines (Mitobe et al., 2009). Like temperature, osmotic pressure is an important signal for full activation of the virulence system (Porter and Dorman, 1994). In both cases, *virB* mRNA turnover is dependent on the RNA chaperone Hfq (Mitobe et al., 2008, 2009). These findings indicate that down-regulation of *virB* gene expression involves rapid turnover of the mRNA when growth conditions no longer correspond to those found in the human large intestine.

The similarity of the VirB protein to ParB-like plasmid partitioning proteins and the similarity between the binding site in DNA used by VirB and those used by ParB-like proteins (*parS*) suggests that the VirB protein is a vestige of a mechanism that was concerned with plasmid segregation and that VirB has been co-opted into gene regulation (Adler et al., 1989; Taniya et al., 2003; Turner and Dorman, 2007). The utility of VirB as a general antagonist of transcriptional silencing mediated by H-NS has been shown by using it to overcome silencing by H-NS of the *proU* operon (Kane and Dorman, 2011). The *proU* operon encodes a transport system for the osmo-protectants glycine-betaine and proline and is not normally controlled by VirB (Ref for ProU). However, judicial placement at the *proU* promoter of the *parS-*like binding site used by VirB at the *S. flexneri icsB* promoter brings *proU* under VirB control. This simple experiment demonstrates the modular nature of gene control mechanisms based on H-NS silencing and anti-silencing and illustrates the ease with which novel systems might evolve in nature.

The VirB protein forms a positive feedback loop onto its own gene’s expression and onto that of the *virF* gene (Kane and Dorman, 2012) (Figure 7). This may facilitate reinforcement of the transition from the ‘off’ to the ‘on’ state once a commitment to expressing the virulence cascade is made. An examination of the fold-induction of the virulence regulon in response to the thermal signal reveals a gearing effect, with *virF* showing the narrowest range of transcription between its induced and non-induced states, the major virulence operons having the widest range and *virB* has an induction ratio with an intermediate value (Porter and Dorman, 1997a). The imposition of additional regulatory signals and regulators may help to restrict full production of the T3SS and its effector proteins to circumstances when the bacterium is most likely to benefit from the physiological investment. This is consistent with findings that reveal roles for post-transcriptional control in the system, such as the phenotypes associated with mutants deficient in *tgt* or *miaA* (Durand and Björk, 2003). The translation control factors elongation factor P (EF-P) and the enzyme that modifies it (PoxA) are also needed for expression of a full virulence phenotype (Marman et al., 2014). It has been suggested that the stability of *virB* mRNA is influenced through interaction with the cytoplasmic-membrane-associated RodZ protein, a determinant of bacterial cell morphology (Mitobe et al., 2011). Other examples of additional control include the sRNA known as RnaG (Tran et al., 2011), the MxiE AraC-like transcription factor, the FNR anaerobic regulator (Marteyn et al., 2010; Vergara-Irigaray et al., 2014), the Rho transcription termination factor (Tobe et al., 1994), the Hfq RNA chaperone (Mitobe et al., 2008, 2009) and the osmo-regulatory OmpR/EnvZ two-component regulatory system (Bernardini et al., 1990, 1993).

## Additional Virulence Regulators in *S. flexneri*

The role of the OmpR/EnvZ system in virulence gene expression seems to be restricted to conditions of high osmolarity growth, but precise molecular details are lacking (Bernardini et al., 1990). The gene encoding the OmpC outer membrane porin is known to require OmpR for expression and has been shown to be required for full virulence, but the *ompC* gene is part of the core genome and is located on the *S. flexneri* chromosome (Bernardini et al., 1993). The possibility that OmpR is involved in the pH response of the *Shigella* virulence system does not seem to have been investigated: OmpR plays such a role in controlling the transcription of T3SS genes in the facultative intracellular pathogen *Salmonella* (Quinn et al., 2014; Chakraborty et al., 2017).

MxiE is encoded by a gene in the Entry Region of the virulence plasmid and plays a role late in the program of virulence gene expression (Kane et al., 2002) (Figures 1, 7). MxiE is an AraC-like transcription factor that operates in partnership with the co-activator IpgC (Pilonieta and Munson, 2008). The IpgC protein is also a chaperone of the IpaB and IpaC effector proteins. MxiE binds to a 17-bp ‘MxiE box’ and regulates several virulence genes on the plasmid and may have targets on the chromosome too (Mavris et al., 2002; Bongrand et al., 2012). Transcriptional slippage by RNA polymerase that introduces an additional base into the MxiE mRNA is required for the production of full-length MxiE protein. The slippage event (and the associated translational frameshift) occurs in about 30% of cases and may represent an additional level of control over *mxiE* gene expression (Penno et al., 2005). This arrangement also influences the efficiency of expression of the immediately-downstream-and-overlapping *mxiD* gene (Penno and Parsot, 2006). Control of MxiE activity is complex and involves a number of other proteins: the anti-co-activators IpaB and IpaC oppose the positive action of the co-activator IpgC by competing with MxiE for IpgC. The anti-activator OspD1, in association with Spa15, inhibits MxiE. The secretion of these co- and anti-activators by the T3SS modulates the activity of MxiE and links its activity to different stages in the operation of the virulence system (Parsot et al., 2005).

Production of the *S. flexneri* T3SS is sensitive to oxygen. The oxygen-sensitive FNR protein, which is related to the cAMP receptor protein (Crp), binds to the large virulence plasmid and represses the transcription of the *spa32* and *spa33* genes (Figure 7). These genes control protein secretion through the T3SS. When the bacterium approaches the epithelial layer at the gut wall, a low concentration of oxygen emanating from the gut surface relieves the FNR-mediated repression, allowing invasion by the bacterium to proceed (Marteyn et al., 2010). In partnership with Fis, Crp regulates the production of the SigA toxin, a cytotoxic protease, encoded by the SHE pathogenicity island on the *S. flexneri* chromosome (Al-Hasani et al., 2001; Rossiter et al., 2015).

## Concluding Remarks

*Shigella flexneri* and *V. cholerae* both cause diarrheal diseases in humans and both employ sophisticated mechanisms for environmental sensing and virulence gene regulation. The sites of the diseases differ in that dysentery is a disease of the lower gut while cholera affects the small intestine and the infection strategies are distinct: host cell invasion (dysentery) and gut lining intoxication (cholera). The infectious dose also differs between the two diseases, being very low for dysentery (about 10 bacterial cells) and much higher for cholera (10^3^ to 10^8^ bacterial cells). These distinctions are consistent with the stronger emphasis on inter-bacterial communication that characterizes *V. cholerae* and evidence that collectives of cells are required at the site of infection for the disease to proceed. It is also consistent with the absence from *V. cholerae* of an extreme acid resistance (XAR) system, a system that *S. flexneri* possesses. The probability that *V. cholerae* can establish an infection is reduced if large numbers of bacteria are killed in the low pH environment of the stomach, creating the need for a large initial inoculum when the host ingests the organisms (Lund et al., 2014). Both pathogens rely on mobile genetic elements that encode virulence factors: pathogenicity islands, a large mobilizable plasmid (dysentery) and a phage (cholera). They share a dependency on the H-NS protein to achieve transcription silencing of their virulence genes, and in the case of *S. flexneri*, this seems to be a strategy for stable maintenance of the genes and for transmitting them vertically. AraC-like transcription factors feature prominently in virulence gene regulation in both organisms, although *V. cholerae* and not *S. flexneri* has a dependence on regulators that are associated with the cytoplasmic membrane. In *S. flexneri*, the key positive regulatory genes are located on the pINV plasmid with their chief target genes; *V. cholerae* uses regulatory genes that are co-imported with the target genes (*toxT*) and others that are part of the core genome (*toxR*). In both species, core-genome members (e.g., Crp, Fnr) play important roles in fine-tuning the regulatory outputs, as do non-H-NS NAPs such as Fis and IHF.

The virulence gene regulatory features shared by *S. flexneri* and *V. cholerae* are also shared by other Gram-negative pathogens, making many of the points raised in this article generally relevant. Horizontal gene transfer has played an important role in the evolution of pathogenic bacteria and the H-NS protein is used widely to silence those genes (Lucchini et al., 2006; Navarre et al., 2006). The mechanisms by which the silencing is overcome in response to specific environmental signals varies from one example to another, but the underlying principle of silencing and anti-silencing of horizontally acquired genes of high A + T base content is a general feature (Stoebel et al., 2008). AraC-like proteins are widespread among Gram-negative bacteria, with many responding to thermal signals and contributing to transcription control in pathogens of mammals (Egan, 2002; Li et al., 2016). Variable DNA topology that responds to environmental stimuli is also widely reported across pathogenic species (Dorman et al., 2016), as are regulatory inputs from nucleoid-associated proteins, inputs that are not restricted to H-NS but include Fis, IHF, HU, and others (Dillon et al., 2010). The specific aspects of virulence gene control in *S. flexneri* and *V. cholerae* are many and reflect the natural histories of those organisms, their lifestyles and infection strategies. This is likely to be true in comparisons of any pathogens, even closely related ones. The general pattern described here, of shared regulatory mechanisms operating as a backdrop for individual, species-specific ones, is likely to hold true across bacteriology.

The research methods used in the field have evolved over the years from those that focus on individual genes and their *cis*- and *trans*-acting regulators to those approaches that survey events across the entire genome (Ayala et al., 2015a, 2017) or employ single-molecule methods (Haas et al., 2015). The result has been both a broader and a deeper understanding of the regulatory events in *V. cholerae* and *S. flexneri*. Whole genome sequencing is revealing important details of the epidemiology of the diseases that these pathogens cause and their evolution (Domman et al., 2017, 2018). It is anticipated that our understanding of the contributions of gene regulatory circuits to their evolution and geographical spread will become ever clearer in the near future.

## Author Contributions

MD and CD researched the topic, wrote the manuscript, and prepared the figures.

## Conflict of Interest Statement

The authors declare that the research was conducted in the absence of any commercial or financial relationships that could be construed as a potential conflict of interest.

## Abbreviations

7PET: seventh pandemic El Tor genetic lineage
CT: cholera toxin
Fis: factor for inversion stimulation
HTH: helix-turn-helix
IHF: integration host factor
NAP: nucleoid-associated protein
pINV: plasmid for invasion
T3SS: type III secretion system
T6SS: type VI secretion system
TCP: toxin co-regulated pilus
VPI-1: *Vibrio* pathogenicity island one
wHTH: winged-helix-turn-helix
